# Prediction of survival and recurrence in patients with pancreatic cancer by integrating multi-omics data

**DOI:** 10.1038/s41598-020-76025-1

**Published:** 2020-11-03

**Authors:** Bin Baek, Hyunju Lee

**Affiliations:** 1 School of Electrical Engineering and Computer Science, Gwangju Institute of Science and Technology, Gwangju, 61005 Korea; 2 Artificial Intelligence Graduate School, Gwangju Institute of Science and Technology, Gwangju, 61005 Korea

**Keywords:** Cancer, Computational biology and bioinformatics

## Abstract

Predicting the prognosis of pancreatic cancer is important because of the very low survival rates of patients with this particular cancer. Although several studies have used microRNA and gene expression profiles and clinical data, as well as images of tissues and cells, to predict cancer survival and recurrence, the accuracies of these approaches in the prediction of high-risk pancreatic adenocarcinoma (PAAD) still need to be improved. Accordingly, in this study, we proposed two biological features based on multi-omics datasets to predict survival and recurrence among patients with PAAD. First, the clonal expansion of cancer cells with somatic mutations was used to predict prognosis. Using whole-exome sequencing data from 134 patients with PAAD from The Cancer Genome Atlas (TCGA), we found five candidate genes that were mutated in the early stages of tumorigenesis with high cellular prevalence (CP). CDKN2A, TP53, TTN, KCNJ18, and KRAS had the highest CP values among the patients with PAAD, and survival and recurrence rates were significantly different between the patients harboring mutations in these candidate genes and those harboring mutations in other genes (*p* = 2.39E−03, *p* = 8.47E−04, respectively). Second, we generated an autoencoder to integrate the RNA sequencing, microRNA sequencing, and DNA methylation data from 134 patients with PAAD from TCGA. The autoencoder robustly reduced the dimensions of these multi-omics data, and the K-means clustering method was then used to cluster the patients into two subgroups. The subgroups of patients had significant differences in survival and recurrence (*p* = 1.41E−03, *p* = 4.43E−04, respectively). Finally, we developed a prediction model for prognosis using these two biological features and clinical data. When support vector machines, random forest, logistic regression, and L2 regularized logistic regression were used as prediction models, logistic regression analysis generally revealed the best performance for both disease-free survival (DFS) and overall survival (OS) (accuracy [ACC] = 0.762 and area under the curve [AUC] = 0.795 for DFS; ACC = 0.776 and AUC = 0.769 for OS). Thus, we could classify patients with a high probability of recurrence and at a high risk of poor outcomes. Our study provides insights into new personalized therapies on the basis of mutation status and multi-omics data.

## Introduction

Pancreatic cancer arises from the abnormal and uncontrolled growth of cells in the tissues of the pancreas. Pancreatic adenocarcinoma (PAAD) is the most common type of pancreatic cancer, accounting for approximately 85% of all types of pancreatic cancer. This cancer is the twelfth most common cancer and the seventh leading cause of cancer-related death. Treatment approaches for pancreatic cancer include surgery, radiotherapy, chemotherapy, and targeted therapy, alone or in combination. The 5-year relative survival rates for pancreatic cancer are 34% when the cancer is only growing in the pancreas, 12% when the cancer has spread to nearby lymph nodes or tissues, and 3% when the cancer has spread to other organs or the lymph nodes^1^. Pancreatic cancer has a high recurrence rate even after treatment, 83.7% (7.8 months of median disease-free survival (DFS)) of pancreatic cancer patients undergoing surgery^2^ and 87% (13.4 months of median DFS) of patients receiving adjuvant chemotherapy after surgical resection^3^ experienced cancer recurrence. Because the overall survival (OS) rates of patients with early tumor recurrence are significantly lower than those of patients without early tumor recurrence^4^, it is necessary to develop novel strategies for predicting recurrence.

The prediction of pancreatic cancer recurrence has been the subject of many studies. Features related to cancer recurrence, such as clinicopathological features^5^, images of tissues and cells^6^, serum CA19-9 levels^7,8^, and gene expression levels (e.g., TP53, bFGF, CD34, and VEGF)^9–11^, have been investigated in previous studies. Because several genes have been shown to be related to pancreatic cancer^12–14^, some studies have considered cancer driver mutations, including those in KRAS, TP53, CDKN2A, and SMAD4, to predict pancreatic cancer recurrence^15^.

Most neoplasms originate from a single cell of origin, and tumor progression results from genetically unstable cells that acquire genetic variants within an original clone^16^, highlighting the importance of understanding tumor progression. In addition, the acquired genetic instability results in individual biological outcomes in the case of advanced human malignant tumors^16^. Andor et al.^17^ classified high-risk cancer patients by inferring clonal expansion but did not investigate cases of pancreatic cancer.

Recently, several types of molecular data have been generated, and many studies have used these data to predict the survival of patients with pancreatic cancer. For example, Xiu et al.^18^ predicted the OS of patients with PAAD using five survival-related microRNA (miRNA) signatures. Additionally, Wu et al.^19^ identified genes that were directly related to pancreatic cancer survival by applying a lasso penalized Cox regression approach for transcriptome analysis. Moreover, Michael et al.^20^ examined the correlations between the survival times of patients with pancreatic cancer and the methylation of individual CpG sites obtained from reduced representation bisulfite sequencing. However, using only one type of molecular data can provide limited information regarding the etiology of tumor progression. Notably, some researchers attempted to classify patients with cancer and predict prognoses using multi-omics data^21,22^. Indeed, Chaudhary et al. predicted survival in patients with liver cancer using a deep learning-based multi-omics model that performed sufficiently well (C-index = 0.70), even without clinical features^23^. Kown et al.^24^ evaluated the diagnostic performance of pancreatic cancer using multiple markers identified based on miRNA and mRNA profiles from 104 pancreatic cancer cases using support vector machine (SVM) modeling and leave-one-out cross-validation. Moreover, Mishra et al.^25^ identified differentially methylated CpG sites and genes correlated with the survival of patients with pancreatic ductal adenocarcinoma using DNA methylation, gene expression, miRNA, and long noncoding RNA expression data. However, these multi-omics data have not been used for the prediction of survival in patients with pancreatic cancer. Multi-omics data can be difficult to use because the high dimensions of the data and the relatively small number of training samples often lead to overfitting. Therefore, learning new features from the multi-omics data that are beneficial to the prediction of prognosis is an important step in the machine learning model-based prediction of survival and recurrence.

In this study, instead of considering only known cancer driver mutations, we considered all somatic mutations from whole-exome sequencing (WES) data obtained from patients with PAAD by inferring the clonal expansion of DNA mutations and selected cancer driver genes. Furthermore, we integrated multi-dimensional genomic data consisting of mRNA, miRNA, and DNA methylation data and then clustered the patients into two subgroups to determine the differences in survival and recurrence between the two groups. Thereafter, we applied several machine learning models to integrate the clonal expansion of DNA mutations and high-dimensional multi-omics data to classify patients based on 5-year DFS and OS.

## Results

### Datasets

**Figure 1:**
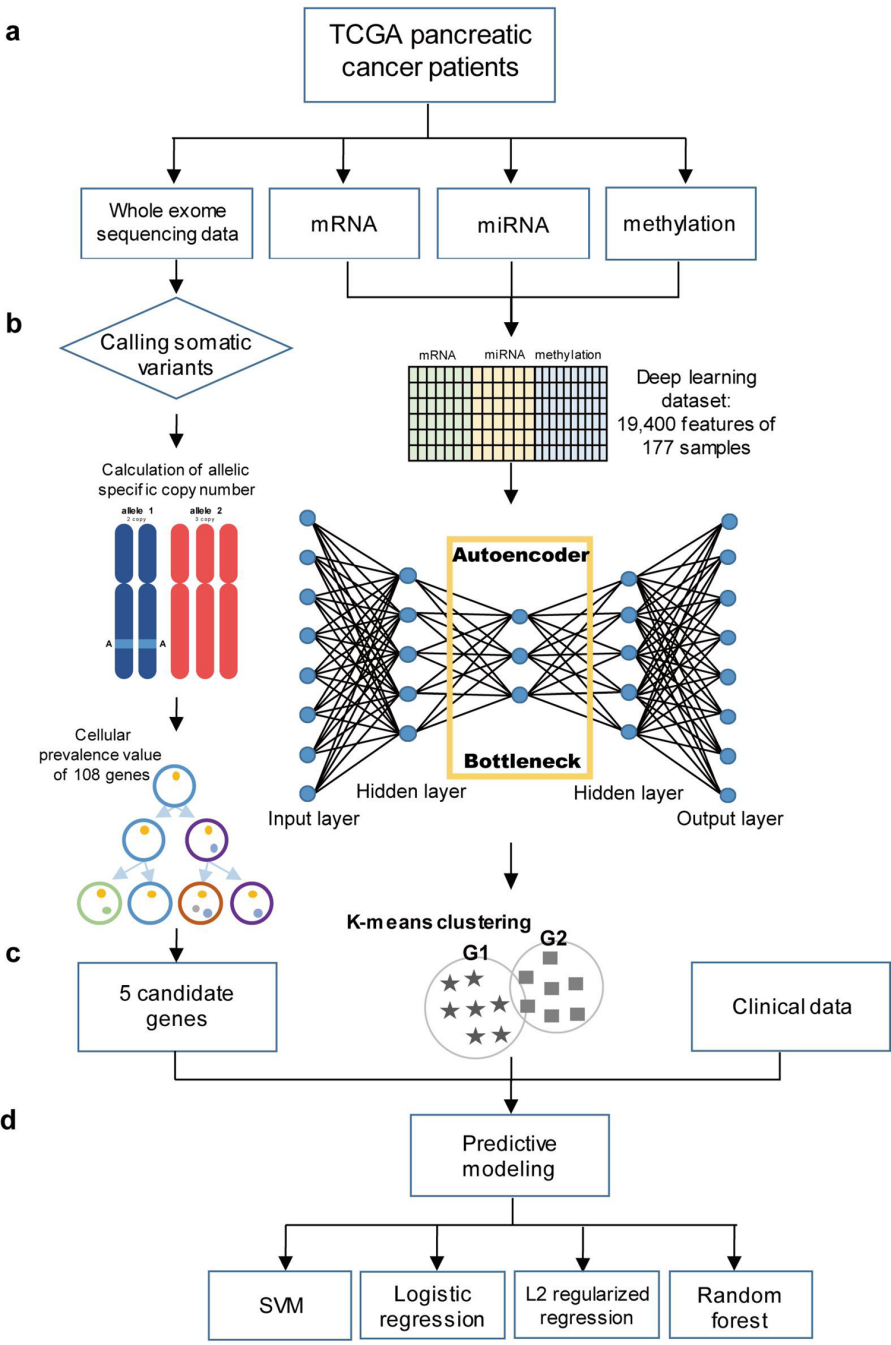
Workflow of approach. Graphical summary of the prediction of survival and recurrence in patients with pancreatic cancer. (**a**) Omics datasets used to construct the prediction models. (**b**) Data preprocessing and the process for obtaining features. (**c**) Final nine features (including seven clinical features). (**d**) Machine learning models used for the prediction.

We used four types of omics data as described in Fig. 1a from The Cancer Genome Atlas (TCGA) (https://portal.gdc.cancer.gov/). Authorization was obtained from the database of Genotypes and Phenotypes (accession No. phs000178.v8.p7). We downloaded WES data from cancer cells and matched normal cells of 183 patients with pancreatic cancer. In addition, we downloaded DNA methylation data from 184 samples, mRNA sequencing data from 177 samples, and miRNA sequencing data from 183 samples using the R package TCGAbiolinks (v.2.10.4)^26^. We were able to obtain clinical data from TCGA cancer samples using the cBioportal (http://www.cbioportal.org/). For multi-omics integration, we used 134 samples with DFS_status and OS_status information, which represents the recurrence and survival of patients, respectively.

### Overview of the approach

We created two biological features from four types of omics data. We called single nucleotide polymorphisms (SNPs) from the WES data, calculated the allele-specific copy numbers of the somatic mutations, and obtained cellular prevalence (CP) values through clones, which is a set of several mutations, illustrated in Fig. 1b. Subsequently, we chose five candidate genes that had the highest CP values, i.e., the first biological feature. For the second feature, we integrated three types of omics data (mRNA, miRNA, and methylation) by building an autoencoder using an unsupervised learning algorithm (Fig. 1b). On the basis of the reduced dimensions of the datasets from the bottleneck of the autoencoder, we divided the patients into two subgroups (Fig. 1c). In total, nine features, including seven features from clinical data, were used to construct machine learning models for the prediction of prognosis in patients with pancreatic cancer (Fig. 1d).

### Relationship between the CP values of mutations and cancer prognosis

**Figure 2:**
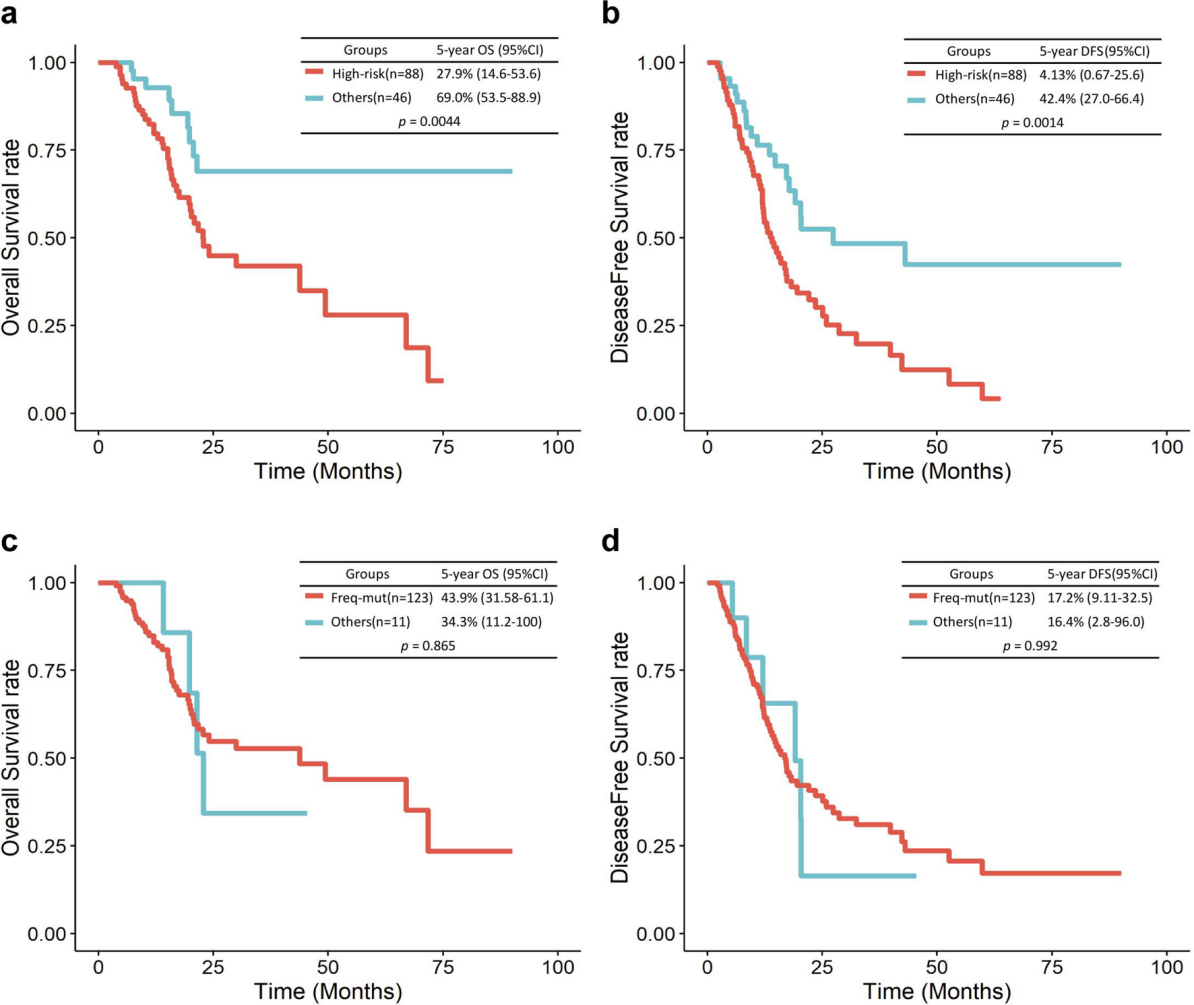
Kaplan–Meier OS and DFS curves for the two groups of patients with PAAD. Kaplan–Meier survival curves for the CP value-based two groups of patients with PAAD. OS (**a**) and DFS (**b**). OS (**c**) and DFS (**d**) of two groups of patients who had mutations in frequently mutated genes and other patients.

CP values were calculated using SNP locations, reference counts, variant counts, allele-specific copy numbers, variant frequencies, and genotypes. A fraction of cancer cells from the variant population was used to determine the CP values of mutations^27^, and the range was between 0 and 1. We finally obtained 2834 SNPs mapped to 108 genes from 7962 SNPs mapped to 3557 genes after excluding genes sharing less than or equal to seven samples (5% of 134 samples). A detailed list of the 108 genes with their CP values is presented in Supplementary Table S1. The top five genes with the highest CP values were CDKN2A (0.722), TP53 (0.667), TTN (0.661), KCNJ18 (0.656), and KRAS (0.655). We defined these five genes as candidate genes. We divided the samples into two groups: 88 samples with mutations in at least one of the five candidate genes and 46 samples without mutations in the candidate genes. We compared cancer recurrence and survival between the two groups on the basis of two indicators, i.e., DFS and OS. We observed that the group with mutations in the candidate genes showed significantly poorer prognosis with regard to DFS (*p* < 0.005, log-rank test; hazard ratio [HR] = 1.744, cox regression) and OS (*p* < 0.005, log-rank test; HR = 1.840, cox regression) than the other group (Fig. 2a,b). Thus, we defined the former group as a high-risk group and the latter as a low-risk group.

To evaluate the predictive power of prognosis for genes with the top five CP values compared with other genes, we further curated five genes that were most frequently mutated (MUC3A, KRAS, TP53, PRSS3, and MUC6) and used these genes as input variables in a Cox regression model. Notably, 123 samples had mutations in at least one of the five frequently mutated genes (Supplementary Table S2). In the Cox regression model, these two groups showed insignificant differences in both DFS and OS (Fig. 2c,d).

### Generation of PAAD subgroups by integrating the mRNA, miRNA, and methylation datasets

From the TCGA-PAAD project, we obtained 134 samples that had mRNA-Seq, miRNA-Seq, and DNA methylation data. For these 134 samples, we preprocessed the data as described in the “Methods”. We obtained 280 of 56,716 genes from the mRNA-Seq, 413 of 1450 miRNAs from the miRNA-Seq, and 18,707 of 374,146 genes from the DNA methylation data (Table 1).

**Table 1:** Statistical analysis of the TCGA-PAAD omics datasets.

	mRNA	miRNA	DNA methylation
Initial number	56,716	1450	374,146
After preprocessing	280	413	18,707

**Figure 3:**
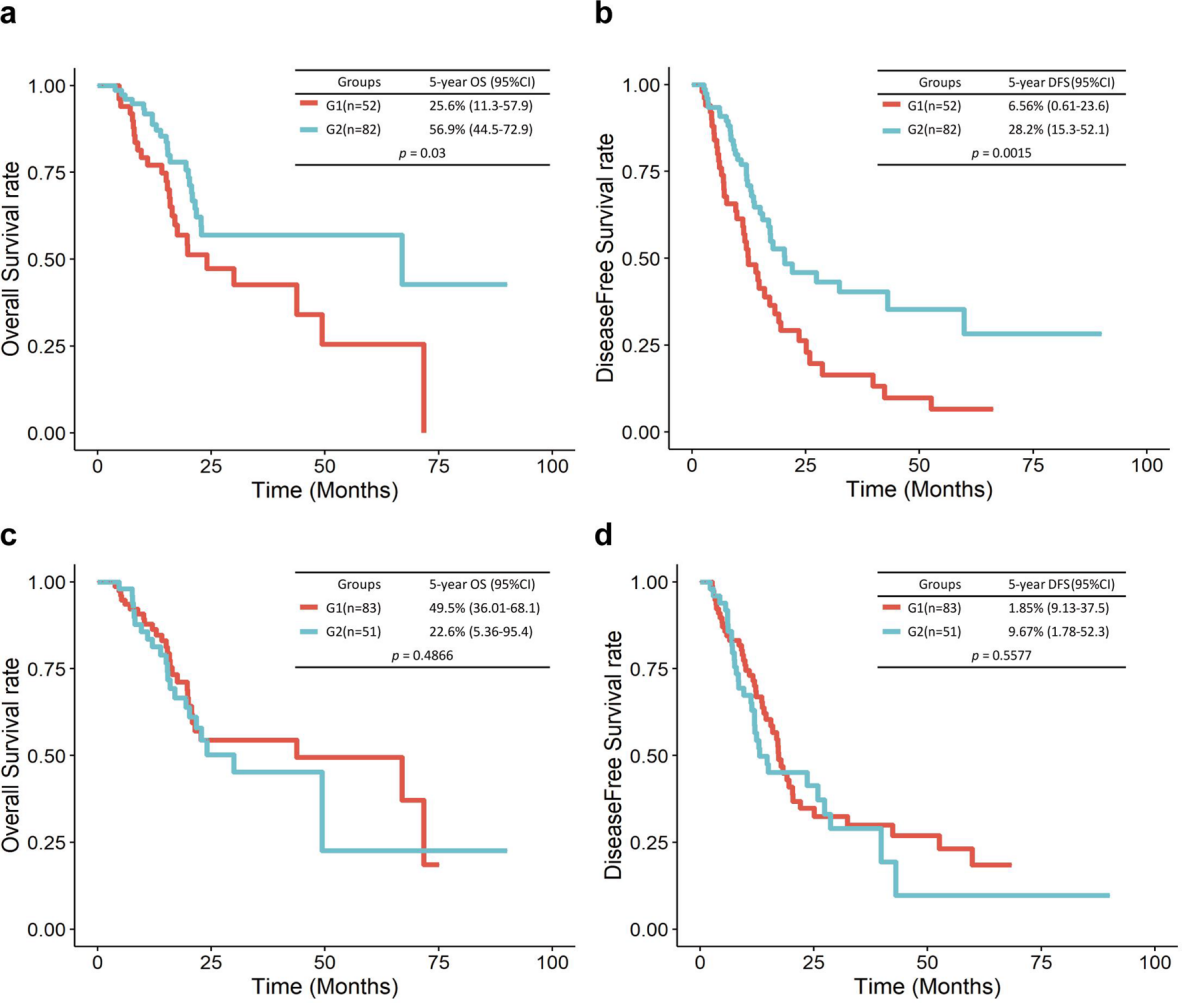
Kaplan–Meier OS and DFS curves for the two groups of patients identified by K-means clustering. Kaplan–Meier survival curves for the two subgroups of patients showing OS and DFS. DFS for G1 and G2 (**a**), OS for G1 and G2 (**b**). Kaplan–Meier survival curves for the two subgroups analyzed by PCA showing OS and DFS. DFS for G1 and G2 (**c**), OS for G1 and G2 (**d**).

The three types of omics data were integrated using an autoencoder. The structure of the autoencoder is shown in Fig. 1b. We obtained 100 new features from the bottleneck hidden layer and used these features as an input for K-means clustering. As a result of clustering, patients were divided into two subgroups, i.e., G1 and G2. G1 and G2 included 52 and 82 patients, respectively. Log–rank survival test was performed to compare the differences between the two groups. Subgroup G1 showed worse prognosis in terms of DFS and OS. The OS rate of patients in G1 was significantly lower than that of patients in G2 (*p* = 0.03; Fig. 3a), and similar results were observed for DFS (*p* = 1.5E−03; Fig. 3b). When we repeated this process 100 times (the dimensional reduction of the three datasets and clustering) and predicted survival and recurrence, 79 of the 100 results showed a significant difference in OS (average *p* = 0.032) and 98 of the 100 results showed a significant difference in DFS (average *p* = 0.012). For comparison, when we reduced the dimensions of each mRNA, miRNA, and methylation dataset to 100 using the autoencoder and divided them into two groups by K-means clustering, survival was not significantly different between the two groups. For mRNAs, the *p* values of OS and DFS were 0.5 and 0.7, respectively. For miRNAs, the *p* values of OS and DFS were 0.4 and 0.2, respectively. For methylation, when the significance of OS and DFS was measured 100 times, 62 times were significant, with an average *p* value of 0.063, for OS, and 67 times were significant, with the average *p* value of 0.056, for DFS).

In addition, we compared the performances of the autoencoder and principal component analysis (PCA). The autoencoder-based classification was superior to the alternative approach. When multi-omics data were reduced using PCA and the samples were divided into two groups by K-means clustering using these reduced data, the two groups do not show differences in DFS or OS (Fig. 3c,d). This result was consistent with the previous observation that PCA could not easily capture the nonlinear relationships present in complex biological data^28^.

### Prediction of the recurrence and survival using machine learning models

To predict the DFS_STATUS and OS_STATUS of patients with PAAD, machine learning models used two biological features and nine clinical features. The two biological features were i) whether a sample had mutations in candidate genes (1 when at least one of the top five genes harbored a mutation and 0 when none of them harbored mutations) and ii) subgroups (G1 or G2) of samples assigned by K-means clustering, integrating mRNA, miRNA, and DNA methylation data. To select clinical features associated with survival status, a stepwise logistic regression analysis was performed with all 14 clinical data (features with missing values in more than 20% of patients were excluded). Seven clinical features including sex, grade, AJCC cancer stage, smoking history, treatment outcome, age, and the primary site were significant for survival status. The features excluded here are indicated in Supplementary Table S3. Along with the seven features, we considered two additional clinical features that were considered potentially important for pancreatic cancer prognosis: treatment (adjuvant radiotherapy) and medical history for diabetes.

**Figure 4:**
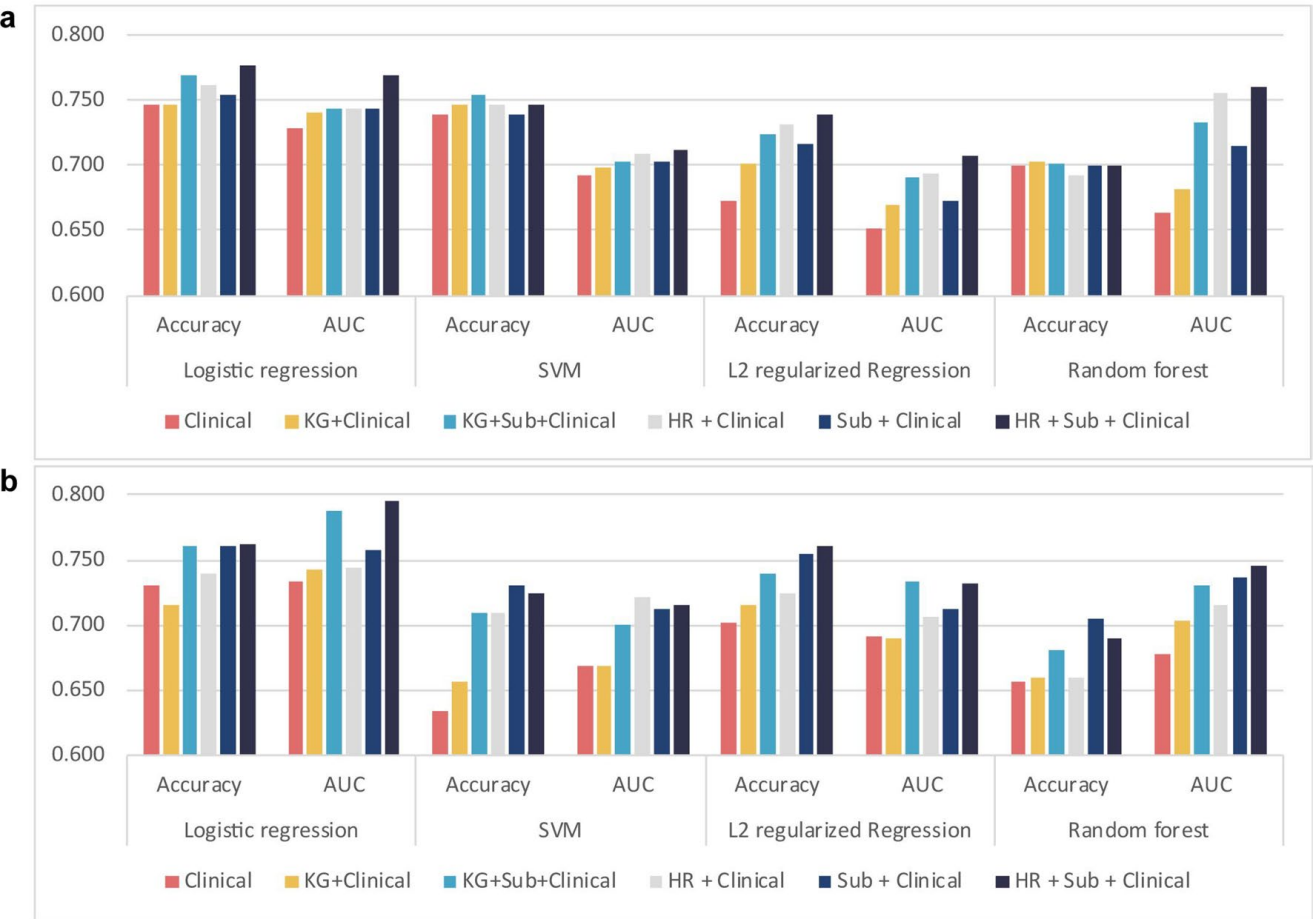
Predictive performance for DFS and OS using various features. The performance of machine learning models for predicting (**a**) OS and (**b**) DFS based on various features were measured. The *y*-axis represents the accuracy or AUC values. Clinical, nine clinical data; KG, known cancer driver genes (KRAS, CDKN2A, TP53, and SMAD4); HR, a high-risk group of patients harboring mutations in five genes with high CP values; Sub, a feature representing subgroups generated by integrating mRNA, miRNA, and DNA methylation subtypes; AUC, area under the curve values from fivefold cross-validation.

Among the prediction models, logistic regression generally showed the best performance for both DFS and OS (accuracy [ACC] = 0.762 and area under the curve [AUC] = 0.795 for DFS; ACC = 0.776 and AUC = 0.769 for OS). The AUCs for OS of SVM, random forest, and L2 regularized logistic regression were 0.711, 0.760, and 0.707, respectively. When all features of biological and clinical data were used in the model, the prediction accuracies of OS and DFS were 3% and 3.1% higher than when only clinical data were used, respectively (Fig. 4). In the previous study^15^, known cancer driver genes including KRAS, CDKN2A, TP53, and SMAD4 were associated with the progression of pancreatic cancer. Among these four genes, three were also included in our top five genes. When we compared the predictive performance for the prognosis of cancer patients using these known driver genes to that using the top five genes in our study, our model displayed higher accuracies and AUC values across most classifiers (Fig. 4).

Furthermore, we used the concordance index (C-index) and the integrated Brier score (IBS) as measures of the accuracy of survival prediction models; the former generalizes the AUC values, and the latter analyzes the average weighted square distance between observed and predicted survival. Thus, models with a high C-index and a low IBS value demonstrate improved accuracy^29^. Table 2 shows that the proposed model using the clinical features and the two binary features outperformed other models on using logistic regression as a classifier.

**Table 2:** Predictive performance for DFS and OS using various features based on C-index and IBS.

Features	C-index		IBS	
	OS	DFS	OS	DFS
Clinical	0.7955±0.04	0.7894±0.04	0.338	0.319
KG+Clinical	0.8019±0.04	0.7937±0.04	0.349	0.317
KG+Sub+Clinical	0.8057±0.04	0.8363±0.03	0.326	0.283
HR+Clinical	0.8152±0.04	0.8125±0.04	0.329	0.308
Sub+Clinical	0.8026±0.04	0.8361±0.03	0.329	0.286
HR+Sub+Clinical	${0.8157}\pm {0.04}$	${0.8388}\pm {0.03}$	0.318	0.265

Logistic regression was used as a prediction model. Clinical, nine clinical data; KG, known cancer driver genes (KRAS, CDKN2A, TP53, and SMAD4); HR, a high-risk group of patients harboring mutations in five genes with high CP values; Sub, a feature representing subgroups generated by integrating mRNA, miRNA, and DNA methylation subtypes.

## Discussion

In a previous study, Andor et al.^17^ calculated the CP values for mutations across 12 cancer types using WES data. When they compared the prognosis of individuals with mutations in genes from the top 5% of CP values (larger clones) and those with mutations in genes from the bottom 5% of CP values (smaller clones), genes mutated in smaller clones predicted poor prognosis across several cancer types (log-rank test, *p* = 2.9E−04; HR = 2.72). However, the researchers did not analyze pancreatic cancer. In this study, we demonstrated that mutated genes with low CP values (mutated in smaller clones) were not significantly related to recurrence and survival. Instead, genes with the top five CP values, typically mutated in the early stages of tumor progression, were predicted to be associated with poor prognosis in PAAD. The five candidate genes identified in this study were CDKN2A, TTN, TP53, KCNJ18, and KRAS. TP53 and KRAS are well-known mutational drivers of pancreatic cancer^30^. Moreover, germline mutations in CDKN2A were reported in pancreatic cancer families^31^. KCNJ18 encodes a member of the inwardly rectifying potassium channel family. Although no previous reports have shown that this gene is related to pancreatic cancer, KCNJ18 has been shown to be involved in esophageal squamous cell carcinoma^32^. The TTN gene has not been studied extensively as a cancer-related gene in the literature but ranked third in our list. This gene provides instructions for generating a giant protein known as titin, which is the largest human protein. In our data, 19 patients had 20 mutations in TTN. Among them, 10 patients died within 5 years (52.6%) and 13 patients showed recurrence within 5 years (68.4%). Because the role of TTN in cancer is unknown^33^, we redrew the Kaplan–Meier OS and DFS curves with a new set of genes, where TTN was excluded from the five candidate genes, and HYDIN, which had the sixth highest CP value, was included as a candidate gene. The result was still significant in both OS (*p* = 0.0158; HR = 2.029) and DFS (*p* = 0.0097; HR = 1.797) (Supplementary Fig. S1a,b). Although HYDIN is not known to be associated with pancreatic cancer, Laske et al.^34^ showed that HYDIN protein is a novel cancer-related antigen recognized by the adaptive immune system.

Furthermore, we determined the predictive power for recurrence and survival, using different numbers of top genes (3, 5, 7, 10, 20, 50, and 100 genes) with the highest CP values (Supplementary Table S4). Among the cases with less than 10 genes, patients harboring mutations in at least one of the top genes had a poorer prognosis with regard to DFS and OS than those not harboring any mutations. However, as shown in Supplementary Table S4, an increase in the number of genes increased the proportion of patients belonging to group 1 (a group of patients harboring mutations in the top genes) because genes with the highest CP values are generally mutated early during tumorigenesis and mutations were widely spread among numerous patients. For example, for the top 10 genes, along with the aforementioned top five genes, HYDIN, HLA-C, HLA-DRB1, MUC5AC, and CES1 were added to the candidate gene list. There were 110 patients with mutations in at least one of these 10 candidate genes, whereas 24 patients did not have any mutations. When we drew the Kaplan–Meier curves to identify differences in OS and DFS between the two groups, we obtained significant results (*p* = 0.04) for OS prediction but not for DFS prediction (*p* = 0.1; Supplementary Fig. S1c,d). For the top 20, 50, and 100 genes, no significant difference in OS and DFS prediction were noted.

In the “Results” section, to represent patients harboring mutations in the candidate genes in machine learning models for predicting recurrence and survival, we used a single binary feature with a value of 1 (a high-risk group) when at least one of the candidate genes was mutated and 0 when none of them were mutated. Instead of using this single binary feature, we also considered using a one-hot vector with the size of candidate genes, where each gene has a value of 1 if mutated. Supplementary Table S5 compares prediction models using a single binary feature and a one-hot vector when the top five and seven candidate genes were used. A logistic regression model, displaying the best performance among the prediction models indicated in Fig. 4, was used. To assess the accuracy and AUC of DFS and the accuracy of OS, the single binary feature representation outperformed the one-hot vector representation.

Furthermore, when preprocessing each omics data before integrating them, probes with low variance in DNA methylation data were filtered out; however, variance-based filtering was not performed for mRNA and miRNA data. To investigate the effects of probes with low variance in mRNA and miRNA data, we used probes with high variance in mRNA and miRNA data. Supplementary Table S5 compares the use of probes with 1% high variance in mRNA and miRNA data, those with 2% high variance, and those with no variance-based filtering. The performance upon using probes without variance-based filtering for mRNA and miRNA data was optimal when the top five genes represented with a single binary feature were integrated. Together, Supplementary Table S5 displays all combinations of using the top three, five, and seven candidate genes with mutations, using a single binary feature and an one-hot vector of mutations, and using mRNA and miRNA data with or without variance-based filtering. Among these combinations, the use of the top five candidate genes harboring mutations in a single binary feature representation and using probes without variance-based filtering in mRNA and miRNA displayed optimal performance.

Additionally, we validated the five candidate genes using another dataset. We obtained 386 PACA-AU samples with clinical data from the International Cancer Genome Consortium (ICGC) (https://dcc.icgc.org/). Among the five candidate genes, the numbers of samples with mutations in TP53 and KRAS were 240 and 345, respectively, and there were 361 samples with mutations in either gene. Because the fraction of samples with mutations in two genes was too large, we used the other three genes (CDKN2A, TTN, and KCNJ18) to divide samples into two groups depending on whether the sample had mutations in at least one of the three genes. There were 121 samples with mutations. The difference in OS between the two groups in the Kaplan–Meier curves was insignificant (*p* = 0.08; Supplementary Fig. S2a). For DFS, the curves of the two groups showed significant differences (*p* = 0.02; Supplementary Fig. S2c). Similarly, in the TCGA-PAAD dataset, 42 samples had mutations in at least one of the three genes; the OS curve showed insignificant results (*p* = 0.2), whereas a significant result was obtained for DFS (*p* = 0.01; Supplementary Fig. S2b,d online). Of the other three genes, CDKN2A was previously shown to affect pancreatic cancer. Thus, we performed Kaplan–Meier survival analysis to recognize the independent effects of the two unknown genes by identifying differences in OS and DFS between patients with and without mutations in TTN and KCNJ18. In the PACA-AU-ICGC dataset, 65 samples had mutations in TTN and KCNJ18. The analysis of OS and DFS revealed significantly different results between the two groups (*p* = 0.01 and 0.03, respectively; Supplementary Fig. S3a,c online). Similarly, there were 33 samples with mutations in the two genes in the TCGA-PAAD dataset. Although the OS analysis yielded insignificant results (*p* = 0.2), the DFS analysis yielded significant results (*p* = 0.04; Supplementary Fig. S3b,d). On the basis of these results, we assumed that the two genes would affect the recurrence of pancreatic cancer.

We adopted an autoencoder to integrate and reduce the dimensions of the mRNA, miRNA, and DNA methylation data and identified two prognostic subgroups, G1 and G2, in PAAD. For the autoencoder, we used three hidden layers after using one and five hidden layers. When one hidden layer was used, the training data were not appropriately decoded and training loss was not reduced below a certain value. When five hidden layers were used, with a continuous reduction in training loss, the test loss was not reduced, and the prediction results were worse than those on using the three hidden layers. The subtype G1 was associated with worse prognosis than the subtype G2 in terms of both OS and DFS. We obtained 789 and 63 differentially expressed genes (DEGs) in subgroups G1 and G2, with a Benjamini-Hochberg adjusted *p* value of less than 0.05. Using these DEGs, we conducted GO pathway enrichment analysis using DAVID for both subgroups. The aggressive subgroup G1 was enriched in 36 pathways, whereas G2 was only enriched in one pathway. As shown in Supplementary Table S6 and Fig. S4a, DEGs in G1 were enriched in 24 biological process terms, e.g., digestion (GO:0007586), ectoderm development (GO:0007398), and epidermis development (GO:0008544), and 12 cellular component terms (Supplementary Table S6 and Fig. S4b online), e.g., cell junction (GO:0030054) and chromosomal part (GO:0044427). In contrast, the moderate subtype G2 was activated only in the molecular function term of alkaline phosphatase activity (GP:0004035; Supplementary Table S7).

In this study, we generated two biological features related to the prognosis of patients with PAAD, i.e., candidate genes based on the CP of mutations and the integration of mRNA, miRNA, and DNA methylation data. Furthermore, we generated prognostic prediction models for predicting cancer recurrence and survival within 5 years. In our predictive model, we used several different types of omics data. The cost to obtain these data sets in a clinical trial can be high. However, with a marked reduction in the cost of sequencing technology, the present results would be applicable in clinical trials in the near future.

## Methods

### Identification of mutated genes related to survival and recurrence based on clonal expansion

#### Calling high-confidence somatic variants

VarScan2 is a tool for detecting somatic mutations and copy number alterations in exome sequencing data from tumor–normal pairs. It calls somatic variants (SNPs and indels) using a statistical test and a heuristic method based on the number of aligned reads supporting each allele^35^. We called SNPs using the “somatic” option. The threshold for minimum read depth at a position to make a call was less than 3, the minimum variant allele frequency threshold was greater than 0.05, and the *p* value threshold for calling variants was greater than 0.05. We detected the copy number using the “copynumber” option. Subsequently, the “ProcessSomatic” option was used for separating whole SNP data into germline, LOH, and somatic, with a threshold for minimum tumor frequency of greater than 0.08. SNP data that were highly confident among somatic variations were used for the analysis.

#### Calculation of allele-specific copy numbers

Sequenza is a software package that uses paired tumor–normal DNA sequencing data to estimate tumor cellularity and ploidy and calculate allele-specific copy number profiles and mutation profiles^36^. Using the input of highly confident somatic mutations and copy numbers, this tool calculates the major and minor copy numbers, which are required to obtain CP values in the next step. We used the mutation frequency threshold of greater than 0.05 and a threshold for minimum frequency of aberrant type of less than 0.6.

#### Calculation of cellular prevalence values

PyClone is a Bayesian clustering method for grouping sets of deeply sequenced somatic mutations into putative clonal clusters while estimating their CP and accounting for allelic imbalances introduced by segmental copy number changes and normal-cell contamination^27^. Human cancer is driven by genetic changes that alter the molecular forms of individual cells^16^. As a result, tumors often consist of several genetically different populations of cells^37^. From these populations, which are called clones, clonal population structures and the origin of genomic alterations can be inferred^27^. A mutation with a higher CP value can be assumed to be a mutation occurring early in the progression of cancer. We adjusted the CP values with regard to the founder mutation of each sample to adjust the bias between samples. The largest inferred clone in each sample represented a founder mutation, which corresponded to the first (founder) clone expansion^17^.

#### Annotation of genetic variants

Annovar is a software tool that utilizes up-to-date information to functionally annotate genetic variants detected from diverse genomes^38^. This tool requires a list of variants with chromosome, start positions, end positions, reference nucleotides, and observed nucleotides. We used Annovar to obtain a list of gene names for somatic mutations in specific genomic regions identified by VarScan2.

#### Selection of candidate genes related to survival and recurrence and survival analysis

After assigning gene names to somatic mutations, we listed all the mutated genes for all samples and counted the number of samples having variants in each mutated gene. Next, the mean CP value of a gene was calculated. Genes that occurred in less than five samples were removed. The genes were then sorted in descending order according to their CP values. We considered the top five genes with the highest CP values as candidate genes. To classify patients at a high risk of poor survival and recurrence, samples with mutations in at least one of the candidate genes and samples without mutations in any of the candidate genes were divided into two groups. Comparisons of each group were evaluated using the Kaplan–Meier survival curves^39^, log-rank test^40^, and HRs from univariate Cox models^41^ were used to estimate the risk associated with each factor. A Kaplan–Meier survival curve was plotted using the survfit function, and a log-rank test was performed using the survdiff function of survival packages^42^ in R.

### Generation of subgroups of patients by integrating the mRNA, miRNA, and DNA methylation data

#### Preprocessing of mRNA, miRNA, and DNA methylation data

For mRNA data, protein-coding genes were selected from the raw counts of mRNA expression data, and DEGs were identified using the DESeq2 tool^43^. Then, we removed genes whose log2 fold changes were less than $\hbox {log}_2$1.5 and whose adjusted *p* values were greater than 0.05. For both mRNA and miRNA data, genes that had more than 20% zero values among samples were removed, and the expression values of the genes were min–max scaled. For DNA methylation data, we checked that the $\beta$-values of all methylated CpG sites had bimodal distributions, with peaks of hypomethylation and hypermethylation within each sample. The standard deviations of the $\beta$-values were used to select probes with a high variance across the entire PAAD tumor dataset^44,45^. We selected approximately 5% of probes.

#### Data integration by the autoencoder

An autoencoder was used to represent mRNA, miRNA, and DNA methylation data. To train the autoencoder, 177 samples from patients with PAAD were randomly split into training and testing datasets using 80% and 20% of samples, respectively. We used Keras (v.2.1.6)^46^. The training dataset was used to train the weights of the model through a gradient descent algorithm. The performance of the model was cyclically evaluated in the test dataset during training; if validation errors continued to increase for five iterations, early stopping could be applied to prevent overfitting. We implemented an autoencoder with three hidden layers with sizes of 500, 100, and 500 nodes. The model was trained using 100 epochs with 50% dropout, and hyperbolic tangent was used as an activation function. The Adam optimizer was used for optimization, and binary cross entropy was used as a loss function. We used an L2 activity regularizer and L1 weight regularizer with values of 0.0001 and 0.001, respectively. The bottleneck layer with 100 hidden nodes was used to produce new low-dimensional features from the multi-omics data.

#### Detecting subgroups by K-means clustering

The multi-omics data were reduced to a vector with a size of 100 using the autoencoder. After removing patients without DFS_status, this feature was used to cluster patients with PAAD into two subgroups using the K-means clustering algorithm. We used a stats package (v.3.5.2)^47^ in R for clustering. After obtaining the two subgroups by K-means clustering, we compared the Kaplan–Meier survival curves of the groups.

### Prediction models

Machine learning classifiers were used to predict the recurrence and survival of patients with PAAD. Logistic regression, L2 regularized logistic regression, SVM, and random forest models were used as classifiers. The features used for these machine learning classifiers were mutation data, integrated multi-omics data, and clinical data. The package stats and MASS^48^ in R were used to select the clinical features used for prediction. In clinical data from TCGA, if a follow-up for DFS_status and OS_status was completed within 5 years (60 months), events such as recurrence and death were classified to have not occurred. Thus, in the machine learning models, we used information regarding the DFS_status and OS_status from the clinical data of TCGA as recurrence and survival values of patients. We conducted fivefold cross-validation 100 times to estimate the accuracy and AUC of the prediction models.

## Supplementary information

Supplementary information
