# Supplementary material for: Prediction of survival and recurrence in patients with pancreatic cancer by integrating multi-omics data
Source: Sci Rep. 2020 Nov 3;10:18951. doi: 10.1038/s41598-020-76025-1 (PMC7609582; doi:10.1038/s41598-020-76025-1)
Supplement: Supplementary file 1 — Supplementary information [file 41598_2020_76025_MOESM1_ESM.pdf]

Supplementary Material

Prediction of survival and recurrence in patients with pancreatic cancer by integrating multi-omics data

Bin Baek[1], Hyunju Lee[1,2*]

[1] Gwangju Institute of Science and Technology, School of Electrical Engineering and Computer Science, Gwangju, 61005, Korea

[2] Gwangju Institute of Science and Technology, Artificial Intelligence Graduate School, Gwangju, 61005, Korea

*hyunjulee@gist.ac.kr

Figure S1: Kaplan-Meier OS and DFS curves between the two groups with mutations in one of the five genes (KRAS, TP53, CDKN2A, KCNJ18, and HYDIN) or more in TCGA-PAAD dataset.

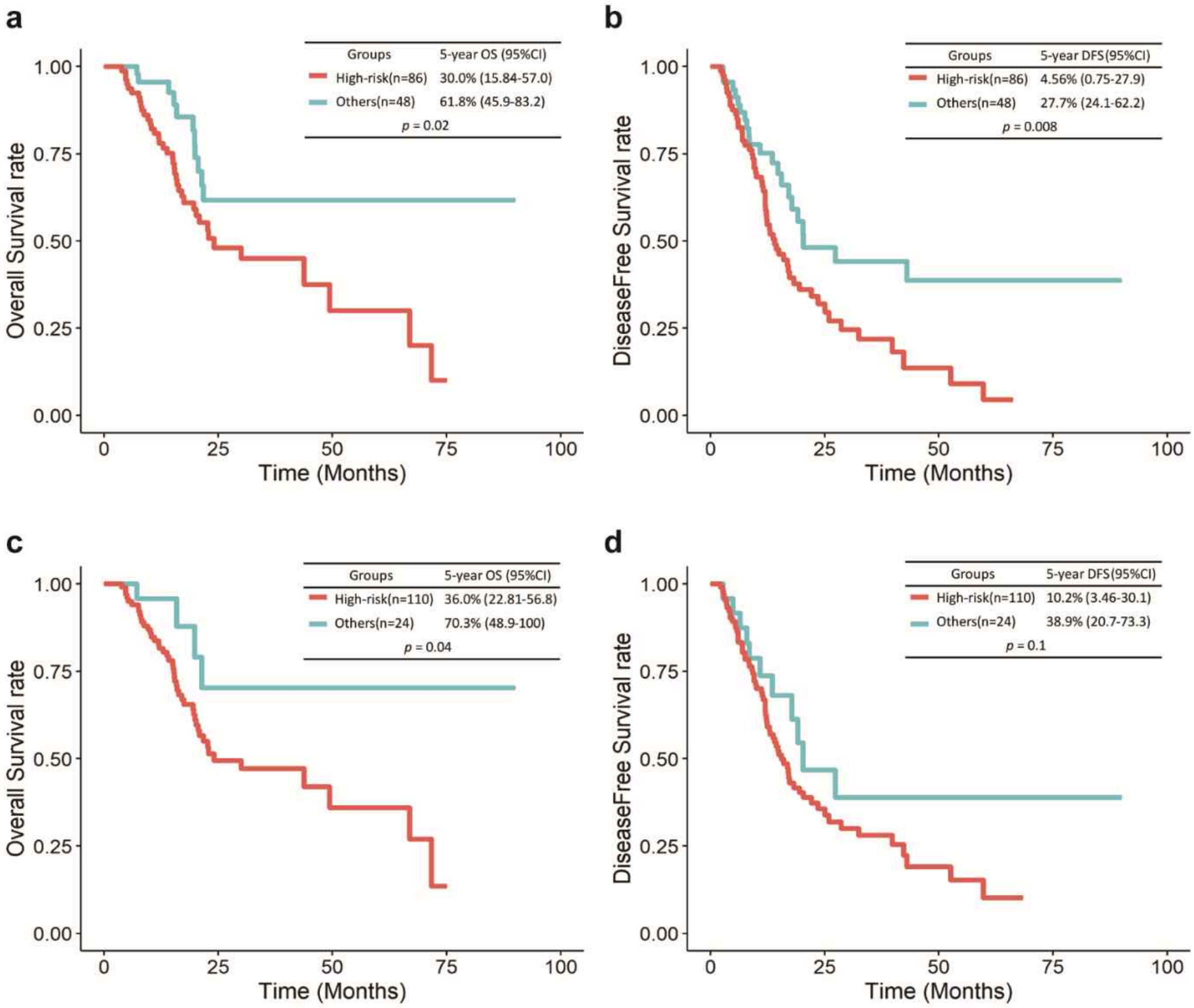

Figure S2: Kaplan-Meier OS and DFS curves between the two groups with mutations in one of the three genes (CDKN2A, TTN, KCNJ18) or more in TCGA-PAAD and PACA-AU-ICGC dataset.

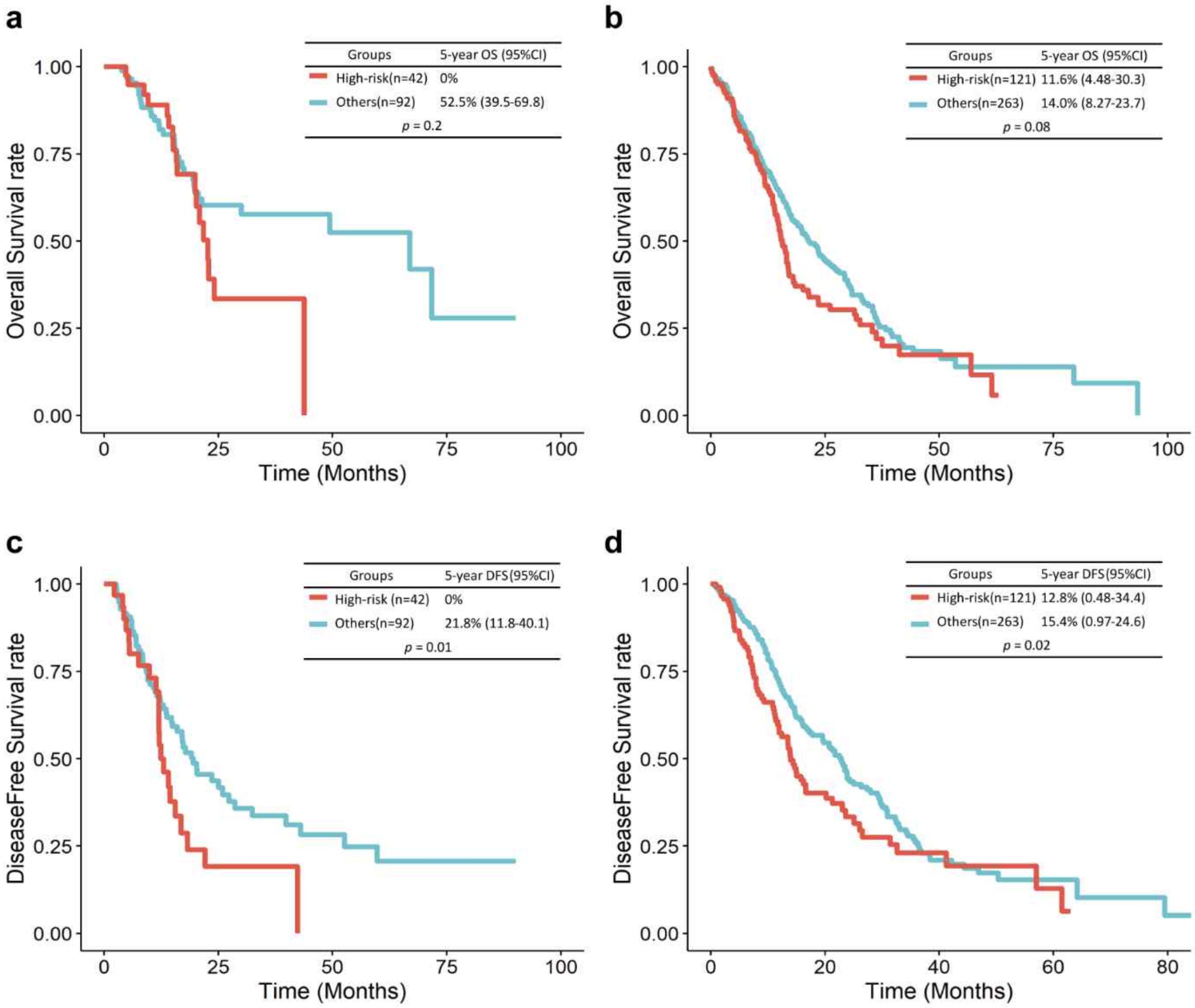

Figure S3: Kaplan-Meier OS and DFS curves between the two groups with mutations in one of the two genes (TTN, KCNJ18) or more in TCGA-PAAD and PACA-AU-ICGC dataset.

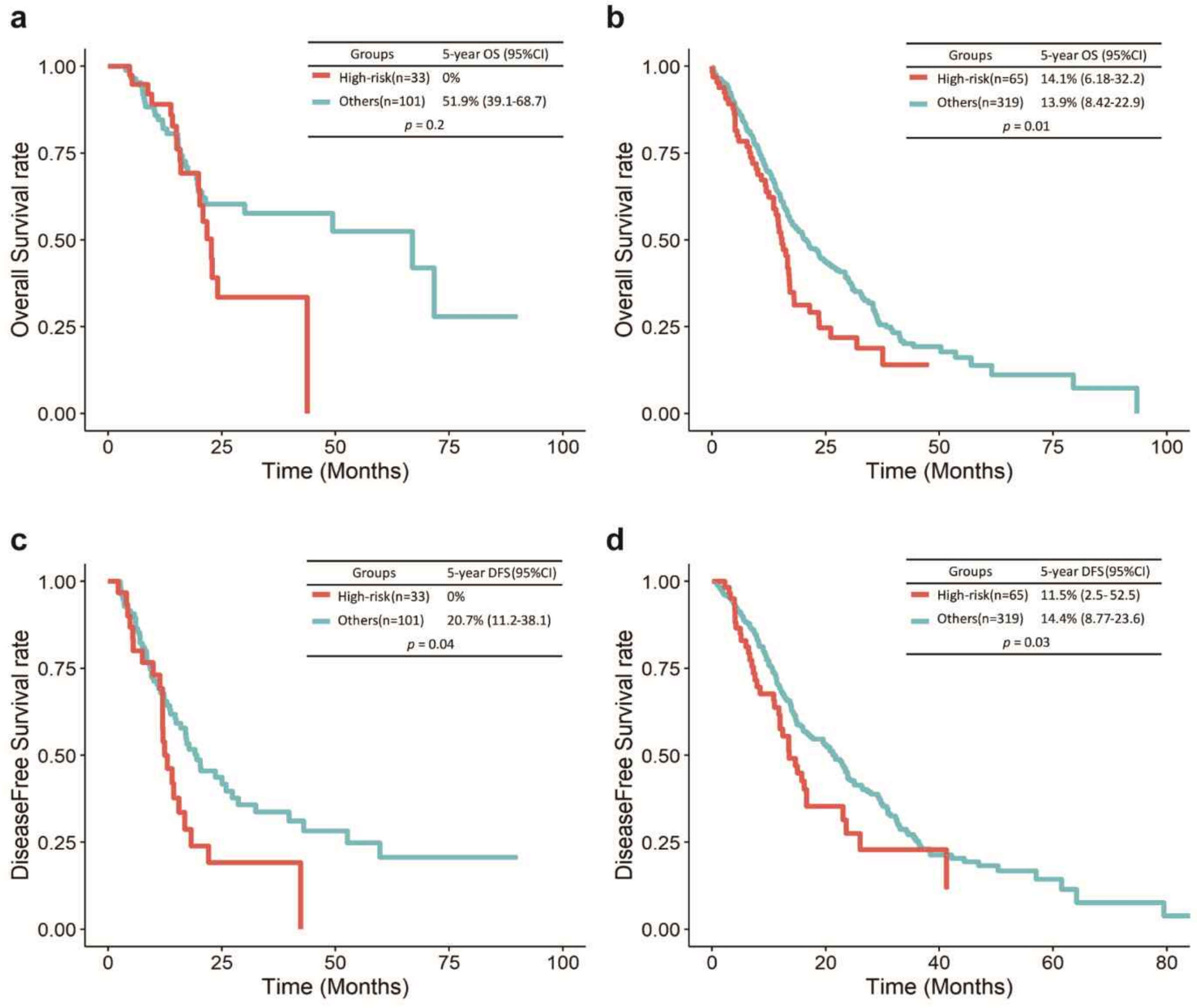

Figure S4: GO pathway analysis in DAVID as an enrichment method for both subtypes G1 and G2 for DEGs.

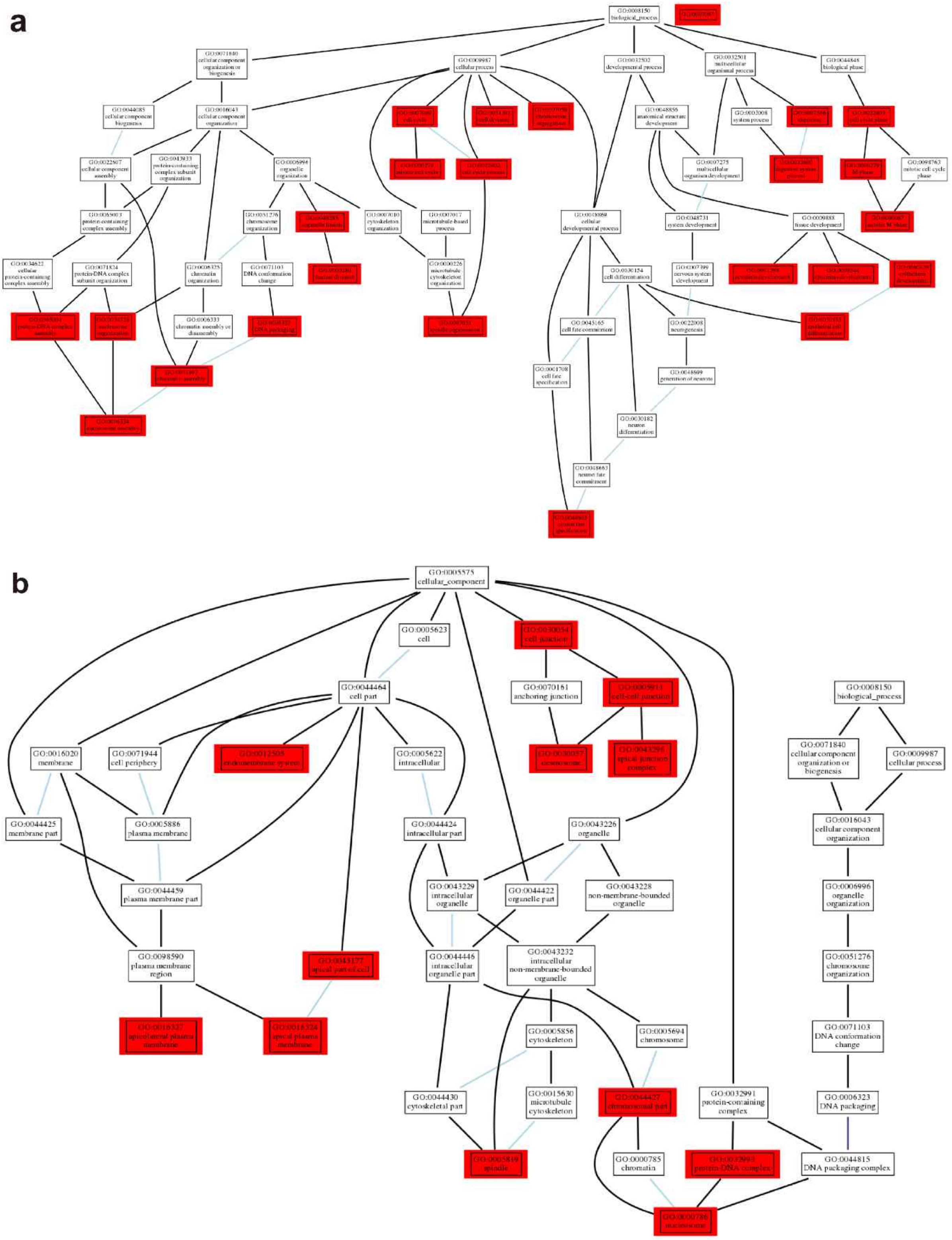

Table S1: List of the 108 genes with CP values

Gene	# Mutation	# Sample	Mean_CP
**CDKN2A**	17	15	0.722009
**TP53**	56	55	0.666956
**TTN**	20	19	0.661295
**KCNJ18**	30	19	0.656332
**KRAS**	68	65	0.654537
**HYDIN**	10	10	0.650837
**HLA-C**	16	10	0.646462
**HLA-DRB1**	51	27	0.645534
**MUC5AC**	25	17	0.639289
**CES1**	20	8	0.628978
**HLA-DQB1**	33	9	0.624154
**EIF4EBP1**	29	8	0.612432
**KRTAP10-2**	14	9	0.606361
**MUC20**	10	8	0.598033
**CDRT1**	8	8	0.592816
**FLG**	12	8	0.590854
**POTEF**	9	9	0.586723
**PRSS1**	48	23	0.585673
**SMAD4**	14	13	0.585333
**HS6ST1**	9	9	0.582984
**FCGBP**	19	18	0.577437
**TAS2R46**	36	20	0.561651
**AGAP9**	11	10	0.560375
**AGAP4**	11	9	0.559187
**FMN2**	27	17	0.558578
**LCE1E**	10	10	0.557022
**OR2T8**	16	13	0.550966
**MUC3A**	251	102	0.550861
**RGPD3**	16	16	0.548322
**MST1L**	18	16	0.547167
**NPIPB11**	8	8	0.543527
**ADAMTS7**	19	12	0.542399
**OR2T33**	13	13	0.542337
**C17orf97**	12	11	0.539022
**LILRA1**	46	24	0.53575
**HLA-DRB5**	48	27	0.528119
**PRSS3**	120	42	0.522026

Table S1(Continued)

Gene	# Mut	# Sample	Mean_CP
**CYP2D6**	13	9	0.520032
**KCNJ12**	57	32	0.518968
**GOLGA6L10**	11	9	0.515922
**MUC5B**	18	16	0.511324
**LRP1B**	8	8	0.510439
**TAS2R43**	35	20	0.509516
**MUC16**	26	9	0.506691
**KRT8**	10	8	0.503223
**OR11H12**	12	9	0.502831
**KRTAP5-4**	11	11	0.498157
**TAS2R31**	30	14	0.497081
**KRTAP4-8**	17	13	0.49313
**PRSS2**	47	25	0.487695
**MYO5B**	27	11	0.480788
**KIR2DL4**	23	11	0.47686
**TAS2R19**	58	22	0.474835
**TPTE2**	12	8	0.473647
**SLC9B1**	15	14	0.472504
**HRNR**	25	18	0.470865
**HLA-B**	37	19	0.468114
**KRTAP4-11**	19	17	0.467501
**SLC25A5**	88	31	0.465818
**PABPC3**	98	35	0.463753
**PRB2**	12	10	0.461243
**FAM136A**	19	8	0.456388
**POTEE**	10	8	0.455615
**PABPC1**	35	16	0.450201
**WASHC2C**	13	10	0.449258
**KIR3DL1**	10	8	0.444453
**PAK2**	24	16	0.443356
**CRIPAK**	11	10	0.441638
**PRAMEF15**	10	8	0.440937
**ESRRA**	49	19	0.439359
**MUC6**	74	38	0.43788
**HLA-DQA2**	39	27	0.4377
**HERC2**	11	10	0.434178

Table S1(Continued)

Gene	# Mut	# Sample	Mean_CP
**TMEM14B**	9	8	0.431865
**AHNAK2**	22	18	0.429024
**MTCH2**	18	11	0.428475
**KRTAP5-10**	8	8	0.426654
**LILRB2**	20	10	0.42348
**AK2**	41	15	0.416627
**SLC35G5**	23	16	0.415274
**PLIN4**	16	13	0.41093
**LILRB1**	24	17	0.40993
**NDUFV2**	12	8	0.407734
**TAS2R30**	40	15	0.407581
**HLA-A**	31	16	0.407174
**MAGEC1**	18	17	0.404557
**HSPD1**	10	8	0.398995
**GBP4**	17	9	0.398516
**NASP**	10	10	0.396249
**LILRA2**	10	9	0.380205
**RANBP2**	10	10	0.379179
**KIR3DL2**	19	10	0.378035
**IRS1**	15	11	0.377354
**PRB1**	9	8	0.375501
**ZNF208**	15	13	0.371259
**PARP4**	20	12	0.370569
**KMT2C**	9	9	0.361993
**BAGE3**	11	10	0.358582
**RBMXL1**	28	12	0.356998
**OR8U1**	20	14	0.352719
**SLC35G6**	12	10	0.349981
**PCDHB4**	13	11	0.346215
**RBMX**	21	11	0.344243
**MUC21**	57	17	0.343872
**UBXN11**	12	9	0.340893
**FAM8A1**	23	8	0.335493
**PCDHGA3**	9	8	0.324649
**NUP50**	8	8	0.313037

Table S2: List of the frequently mutated genes

Gene	# Mut	# Sample	Frequency
**MUC3A**	251	102	0.76
**KRAS**	68	65	0.49
**TP53**	56	55	0.41
**PRSS3**	120	42	0.31
**MUC6**	74	38	0.28
**PABPC3**	98	35	0.26
**KCNJ12**	57	32	0.24
**SLC25A5**	88	31	0.23
**HLA-DRB1**	51	27	0.20
**HLA-DRB5**	48	27	0.20
**HLA-DQA2**	39	27	0.20
**PRSS2**	47	25	0.19
**LILRA1**	46	24	0.18
**PRSS1**	48	23	0.17
**TAS2R19**	58	22	0.16
**TAS2R46**	36	20	0.15
**TAS2R43**	35	20	0.15
**TTN**	20	19	0.14
**KCNJ18**	30	19	0.14
**HLA-B**	37	19	0.14
**ESRRA**	49	19	0.14
**FCGBP**	19	18	0.13
**HRNR**	25	18	0.13
**AHNAK2**	22	18	0.13
**MUC5AC**	25	17	0.13
**FMN2**	27	17	0.13
**KRTAP4-11**	19	17	0.13
**LILRB1**	24	17	0.13
**MAGEC1**	18	17	0.13
**MUC21**	57	17	0.13
**RGPD3**	16	16	0.12
**MST1L**	18	16	0.12
**MUC5B**	18	16	0.12
**PABPC1**	35	16	0.12
**PAK2**	24	16	0.12
**SLC35G5**	23	16	0.12
**HLA-A**	31	16	0.12

Table S2(Continued)

Gene	# Mut	# Sample	Frequency
**CDKN2A**	17	15	0.11
**AK2**	41	15	0.11
**TAS2R30**	40	15	0.11
**TAS2R31**	30	14	0.10
**SLC9B1**	15	14	0.10
**OR8U1**	20	14	0.10
**SMAD4**	14	13	0.10
**OR2T8**	16	13	0.10
**OR2T33**	13	13	0.10
**KRTAP4-8**	17	13	0.10
**PLIN4**	16	13	0.10
**ZNF208**	15	13	0.10
**ADAMTS7**	19	12	0.09
**PARP4**	20	12	0.09
**RBMXL1**	28	12	0.09
**C17orf97**	12	11	0.08
**KRTAP5-4**	11	11	0.08
**MYO5B**	27	11	0.08
**KIR2DL4**	23	11	0.08
**MTCH2**	18	11	0.08
**IRS1**	15	11	0.08
**PCDHB4**	13	11	0.08
**RBMX**	21	11	0.08
**HYDIN**	10	10	0.07
**HLA-C**	16	10	0.07
**AGAP9**	11	10	0.07
**LCE1E**	10	10	0.07
**PRB2**	12	10	0.07
**WASHC2C**	13	10	0.07
**CRIPAK**	11	10	0.07
**HERC2**	11	10	0.07
**LILRB2**	20	10	0.07
**NASP**	10	10	0.07
**RANBP2**	10	10	0.07
**KIR3DL2**	19	10	0.07
**BAGE3**	11	10	0.07
**SLC35G6**	12	10	0.07

Table S2(Continued)

Gene	# Mut	# Sample	Frequency
**HLA-DQB1**	33	9	0.07
**KRTAP10-2**	14	9	0.07
**POTEF**	9	9	0.07
**HS6ST1**	9	9	0.07
**AGAP4**	11	9	0.07
**CYP2D6**	13	9	0.07
**GOLGA6L10**	11	9	0.07
**MUC16**	26	9	0.07
**OR11H12**	12	9	0.07
**GBP4**	17	9	0.07
**LILRA2**	10	9	0.07
**KMT2C**	9	9	0.07
**UBXN11**	12	9	0.07
**CES1**	20	8	0.06
**EIF4EBP1**	29	8	0.06
**MUC20**	10	8	0.06
**CDRT1**	8	8	0.06
**FLG**	12	8	0.06
**NPIPB11**	8	8	0.06
**LRP1B**	8	8	0.06
**KRT8**	10	8	0.06
**TPTE2**	12	8	0.06
**FAM136A**	19	8	0.06
**POTEE**	10	8	0.06
**KIR3DL1**	10	8	0.06
**PRAMEF15**	10	8	0.06
**TMEM14B**	9	8	0.06
**KRTAP5-10**	8	8	0.06
**NDUFV2**	12	8	0.06
**HSPD1**	10	8	0.06
**PRB1**	9	8	0.06
**FAM8A1**	23	8	0.06
**PCDHGA3**	9	8	0.06
**NUP50**	8	8	0.06

Table S3: Result of a stepwise logistic regression for selecting clinical features

STEP	DF	DEVIANCE	RESID. DF	RESID. DEV	AIC
All 14 features			98	88.67718	162.6772
- ICD10 classification	4	1.41E+00	102	90.08247	156.0825
- AJCC Metastasis Stage Code	2	3.95E+00	104	94.0362	152.0362
- Prior Cancer Diagnosis Occurrence	1	6.21E-01	105	94.65696	148.657
- Person Neoplasm Status	1	5.27E-05	106	94.65701	146.657
- Chronic Pancreatitis Personal Medical History	1	8.76E-02	107	94.74523	142.7452
- Primary Lymph Node Presentation Assessment	1	3.85E-01	108	95.13052	139.1305
- Race Category	1	1.98E+00	109	97.1116	137.1116

AIC (Akaike Information Criterion) evaluates the relative quality of the model for a given data set and is measured by AIC = $-2\ln(L) + 2k$, where L is the likelihood function and k is the number of parameters in the model. The lower the AIC value, the better the model. When all 14 features were added to the null model ($\beta_{1,\ldots}= 0$), the residual deviance decreased as 88.67718 compared to the deviance of null hypothesis and the AIC at that time was 162.6772. The deviation of the ICD 10 classifications was 1.41, and when using only 13 features after excluding the ICD 10 classification, the residual deviance was reduced by 90.08247, making it a better model. When the same procedure was repeated, the seven features in the table were removed.

Table S4: Overall survival (OS) and disease free survival (DFS) using different numbers of the top genes with high CP values

Genes	OS p-values	Samples (0/1)	Observed (0/1)	DFS p-values	Samples (0/1)	Observed (0/1)
3	0.002	72/62	20/30	0.00051	72/62	35/43
5	0.0044	46/88	9/41	0.0014	46/88	19/59
7	0.008	40/94	8/42	0.0037	40/94	17/61
10	0.04	24/110	4/46	0.1	24/110	11/67
20	0.6	13/121	2/48	0.6	13/121	7/71
50	0.7	2/132	1/49	0.7	2/132	1/77
100	0.7	2/132	1/49	0.7	2/132	1/77

Table S5: Performance of prediction models depending on the representation of genes with mutations (binary or one-hot vector), different numbers of top genes, and filtering of mRNAs and microRNAs with low variances.

	**Without variance cut (dim=19400)**				**0.01 variance cut (dim=19243)**				**0.02 variance cut (dim=19005)**			
	**DFS_acc**	**DFS_Auc**	**OS_acc**	**OS_AUC**	**DFS_acc**	**DFS_Auc**	**OS_acc**	**OS_AUC**	**DFS_acc**	**DFS_Auc**	**OS_acc**	**OS_AUC**
**Top 5 genes (binary)**	**0.762**	**0.795**	**0.769**	**0.776**	0.739	0.741	0.746	0.749	0.731	0.767	0.761	0.744
**Top 3 genes (binary)**	0.754	0.784	0.754	0.760	0.739	0.772	0.739	0.735	0.739	0.756	0.739	0.743
**Top 7 genes (binary)**	0.761	0.780	0.739	0.738	0.746	0.758	0.738	0.743	0.739	0.760	0.724	0.750
**Top 5 genes (one-hot)**	0.746	0.752	0.724	0.738	0.739	0.722	0.709	0.737	0.731	0.742	0.739	0.727
**Top 7 genes (one-hot)**	0.746	0.779	0.731	0.740	0.731	0.726	0.724	0.767	0.724	0.738	0.709	0.725

Table S6: GO pathway analysis for DEGs of subtype G1 using DAVID.

Category	Term	Count	%	P-value	Genes
GOTERM_BP_FAT	GO:0000279~M phase	41	5.718271	5.17E-10	KIF23, PRC1, NEK2, TTK, CHEK1, ANLN, AURKA, CEP55, PTTG1, SPC24, FAM83D, SPC25, RSPH1, OIP5, OVOL1, SKA3, CDCA5, ASPM, TRIP13, EXO1, CDK1, CDC6, MKI67, DLGAP5, TPX2, KIF18A, NUF2, CENPF, CDC20, BIRC5, PBK, HMGA2, CDC25C, UBE2C, TACC2, CCNB1, CCNB2, EREG, PLK1, ZWINT, CKS2
GOTERM_BP_FAT	GO:0022403~cell cycle phase	45	6.276151	4.97E-09	E2F1, KIF23, PRC1, NEK2, TTK, CHEK1, ANLN, AURKA, PTTG1, CEP55, SPC24, FAM83D, SPC25, RSPH1, OIP5, OVOL1, SKA3, CDCA5, ASPM, TRIP13, EXO1, CDK1, CDC6, MKI67, DLGAP5, TPX2, NUF2, KIF18A, CENPF, CDC20, BIRC5, PBK, HMGA2, CDKN3, CDC25C, UBE2C, TACC2, CCNB1, CCND1, CCNB2, EREG, PLK1, ZWINT, CKS2, USH1C
GOTERM_BP_FAT	GO:0007586~digestion	19	2.64993	2.08E-08	MOGAT2, SLC5A1, CAPN9, UGT1A1, MUC6, ABCG8, KCNN4, UGT1A10, UGT1A8, SSTR1, AKR1B10, PRSS3, CTSE, TFF2, TFF3, FABP1, MUC5AC, FABP2, TFF1, PYY, NMU
GOTERM_BP_FAT	GO:0007067~mitosis	30	4.1841	2.17E-08	KIF23, NEK2, AURKA, ANLN, CEP55, PTTG1, SPC24, FAM83D, SPC25, OIP5, SKA3, CDCA5, ASPM, CDC6, CDK1, DLGAP5, TPX2, KIF18A, NUF2, CENPF, BIRC5, CDC20, PBK, HMGA2, UBE2C, CDC25C, CCNB1, CCNB2, PLK1, ZWINT
GOTERM_BP_FAT	GO:0000280~nuclear division	30	4.1841	2.17E-08	KIF23, NEK2, AURKA, ANLN, CEP55, PTTG1, SPC24, FAM83D, SPC25, OIP5, SKA3, CDCA5, ASPM, CDC6, CDK1, DLGAP5, TPX2, KIF18A, NUF2, CENPF, BIRC5, CDC20, PBK, HMGA2, UBE2C, CDC25C, CCNB1, CCNB2, PLK1, ZWINT
GOTERM_BP_FAT	GO:0000087~M phase of mitotic cell cycle	30	4.1841	3.26E-08	KIF23, NEK2, AURKA, ANLN, CEP55, PTTG1, SPC24, FAM83D, SPC25, OIP5, SKA3, CDCA5, ASPM, CDC6, CDK1, DLGAP5, TPX2, KIF18A, NUF2, CENPF, BIRC5, CDC20, PBK, HMGA2, UBE2C, CDC25C, CCNB1, CCNB2, PLK1, ZWINT
GOTERM_BP_FAT	GO:0048285~organelle fission	30	4.1841	5.34E-08	KIF23, NEK2, AURKA, ANLN, CEP55, PTTG1, SPC24, FAM83D, SPC25, OIP5, SKA3, CDCA5, ASPM, CDC6, CDK1, DLGAP5, TPX2, KIF18A, NUF2, CENPF, BIRC5, CDC20, PBK, HMGA2, UBE2C, CDC25C, CCNB1, CCNB2, PLK1, ZWINT
GOTERM_BP_FAT	GO:0022402~cell cycle process	50	6.973501	4.30E-07	KIF23, E2F1, PRC1, NEK2, TTK, ANLN, AURKA, CHEK1, CEP55, PTTG1, SPC24, FAM83D, SPC25, SBDS, OIP5, RSPH1, OVOL1, SKA3, CDCA5, ASPM, TRIP13, EXO1, CDC6, CDK1, MKI67, DLGAP5, TPX2, KIF18A, NUF2, CENPF, CDC20, BIRC5, PBK, HMGA2, RACGAP1, CDKN3, CDC25C, UBE2C, TP73, TACC2, CCNB1, CCND1, CCNB2, EREG, IRF6, PLK1, ZWINT, CKS2, USH1C, ERN2
GOTERM_BP_FAT	GO:0000278~mitotic cell cycle	37	5.160391	1.12E-06	KIF23, E2F1, PRC1, NEK2, TTK, CHEK1, ANLN, AURKA, CEP55, PTTG1, SPC24, FAM83D, SPC25, OIP5, SKA3, CDCA5, ASPM, CDK1, CDC6, DLGAP5, TPX2, KIF18A, NUF2, CENPF, BIRC5, CDC20, PBK, HMGA2, CDKN3, UBE2C, CDC25C, CCNB1, CCND1, CCNB2, PLK1, ZWINT, USH1C

Table S6(Continued)

Category	Term	Count	%	PValue	Genes
GOTERM_BP_FAT	GO:0007398~ectoderm development	25	3.48675	1.84E-06	ZBTB7B, KRT6A, CRABP2, GJB3, KRT13, GJB5, EDAR, SFN, SPINK5, SHH, SCEL, FOXQ1, LAMA3, EVPL, EREG, CASP14, IRF6, KRT16, LHX1, PPL, OVOL1, SPRR3, AHNAK2, LAMC2, IVL
GOTERM_BP_FAT	GO:0060429~epithelium development	27	3.76569	1.84E-06	HOXA11, TFCP2L1, SFN, SPINK5, SHH, ZIC2, EZR, OVOL2, HOXA5, PPL, AHNAK2, IVL, UPK2, F11R, TBX4, FOXA1, MECOM, SCEL, LAMA3, EVPL, RHCG, EREG, CA9, IRF6, ZIC5, SPRR3, DMBT1
GOTERM_BP_FAT	GO:0051301~cell division	31	4.32357	3.65E-06	KIF23, CKS1B, PRC1, NEK2, ANLN, CEP55, PTTG1, SPC24, FAM83D, SPC25, OIP5, SKA3, CDCA5, ASPM, CDK1, PARD6B, CDC6, NUF2, CENPF, BIRC5, CDC20, HMGA2, RACGAP1, UBE2C, CDC25C, CCNB1, CCND1, CCNB2, PLK1, ZWINT, CKS2
GOTERM_BP_FAT	GO:0007059~chromosome segregation	15	2.09205	4.23E-06	NEK2, DLGAP5, NUF2, KIF18A, CENPF, BIRC5, PTTG1, SRPK1, SPC25, HJURP, RIOK3, ZWINT, SKA3, CDCA5, TOP2A
GOTERM_BP_FAT	GO:0007049~cell cycle	59	8.228731	4.68E-06	E2F1, KIF23, S100A6, PRC1, E2F7, TTK, AURKA, PTTG1, FAM83D, CDC45, SBDS, RSPH1, OIP5, CDCA5, ASPM, CDK1, CDC6, TPX2, PBK, HMGA2, UBE2C, TACC2, CCND1, EREG, ZWINT, MAPK3, USH1C, ERN2, CKS1B, NEK2, LIN9, FOXM1, ANLN, CHEK1, CEP55, SPC24, SPC25, HJURP, OVOL1, SKA3, TRIP13, EXO1, PARD6B, MKI67, DLGAP5, NUF2, KIF18A, CENPF, BIRC5, CDC20, RACGAP1, CDKN3, CDC25C, TP73, CCNB1, CCNB2, PLK1, IRF6, CKS2
GOTERM_BP_FAT	GO:0065004~protein-DNA complex assembly	15	2.09205	1.70E-05	HIST1H2AC, HIST1H2BC, HIST1H2BD, HIST1H1C, CENPF, HIST2H4A, HIST1H2BK, HIST2H2BE, HJURP, HIST2H2BF, HIST1H2BJ, HIST3H2A, HIST1H4I, ASF1B, HIST3H2BB, HIST1H3H, HIST1H4H
GOTERM_BP_FAT	GO:0008544~epidermis development	22	3.06834	1.89E-05	CRABP2, GJB3, KRT13, GJB5, SFN, EDAR, SPINK5, SHH, SCEL, FOXQ1, LAMA3, EVPL, EREG, IRF6, CASP14, KRT16, PPL, OVOL1, SPRR3, AHNAK2, LAMC2, IVL
GOTERM_BP_FAT	GO:0034728~nucleosome organization	15	2.09205	2.20E-05	HIST1H2AC, HIST1H2BC, HIST1H2BD, HIST1H1C, HIST2H4A, HMGA1, HIST1H2BK, HIST2H2BE, HJURP, HIST2H2BF, HIST1H2BJ, HIST3H2A, HIST1H4I, ASF1B, HIST3H2BB, HIST1H3H, HIST1H4H
GOTERM_BP_FAT	GO:0006334~nucleosome assembly	14	1.95258	3.18E-05	HIST1H2AC, HIST1H2BC, HIST1H2BD, HIST1H1C, HIST2H4A, HIST1H2BK, HIST2H2BE, HJURP, HIST2H2BF, HIST1H2BJ, HIST3H2A, HIST1H4I, ASF1B, HIST3H2BB, HIST1H3H, HIST1H4H
GOTERM_BP_FAT	GO:0030855~epithelial cell differentiation	18	2.51046	4.02E-05	F11R, TFCP2L1, FOXA1, SFN, SPINK5, SCEL, EZR, LAMA3, EVPL, RHCG, EREG, IRF6, PPL, SPRR3, AHNAK2, IVL, UPK2, DMBT1
GOTERM_BP_FAT	GO:0031497~chromatin assembly	14	1.95258	4.65E-05	HIST1H2AC, HIST1H2BC, HIST1H2BD, HIST1H1C, HIST2H4A, HIST1H2BK, HIST2H2BE, HJURP, HIST2H2BF, HIST1H2BJ, HIST3H2A, HIST1H4I, ASF1B, HIST3H2BB, HIST1H3H, HIST1H4H
GOTERM_BP_FAT	GO:0022600~digestive system process	9	1.25523	5.05E-05	ABCG8, KCNN4, MOGAT2, SLC5A1, FABP1, TFF1, FABP2, MUC6, NMU
GOTERM_BP_FAT	GO:0006323~DNA packaging	16	2.23152	7.76E-05	HIST1H2AC, HIST1H2BC, HIST1H2BD, HIST1H1C, HIST2H4A, HIST1H2BK, HIST2H2BE, HJURP, HIST2H2BF, HIST1H2BJ, HIST3H2A, HIST1H4I, ASF1B, HIST3H2BB, CDCA5, TOP2A, HIST1H3H, HIST1H4H

Table S6(Continued)

Category	Term	Count	%	PValue	Genes
GOTERM_BP_FAT	GO:0048665~neuron fate specification	6	0.83682	2.33E-04	HOXC10, EVX1, NKX6-2, FOXA1, HOXD10, SHH
GOTERM_BP_FAT	GO:0007051~spindle organization	9	1.25523	4.04E-04	KIF23, SPC25, PRC1, ZWINT, CKS2, TTK, AURKA, UBE2C, TACC2
GOTERM_CC_FAT	GO:0005911~cell-cell junction	26	3.62622	2.82E-07	CLDN18, SLC5A1, LMO7, GJC2, CLDN12, PCDH1, MARVELD2, PPL, SLC2A1, F11R, PARD6B, STX3, GJB3, GJB5, EVPL, ITGA6, CGN, DSG3, PKP3, PKP4, CDC42BPA, DSC3, TJP3, PERP, PDZD3, STEAP1
GOTERM_CC_FAT	GO:0005819~spindle	21	2.92887	2.59E-06	KIF14, KIF23, CDC6, CDK1, KIF4A, PRC1, NEK2, DLGAP5, TPX2, KIF18A, CENPF, TTK, AURKA, CDC20, BIRC5, RACGAP1, FAM83D, SBDS, PLK1, SKA3, ASPM
GOTERM_CC_FAT	GO:0045177~apical part of cell	23	3.20781	4.59E-06	MUC1, PARD6B, MYO1A, SLC22A18, STX3, CLCA4, ERBB3, SLC5A1, PRKCI, AQP5, DUOX1, LMO7, EDAR, AMN, ABCG8, EZR, RHCG, USH1C, STK39, FABP1, FABP2, SLC39A4, PDZD3
GOTERM_CC_FAT	GO:0043296~apical junction complex	15	2.09205	5.32E-05	F11R, PARD6B, CLDN18, CLDN12, EVPL, CGN, MARVELD2, DSG3, PPL, PKP3, PKP4, DSC3, PERP, TJP3, PDZD3
GOTERM_CC_FAT	GO:0000786~nucleosome	12	1.67364	4.71E-05	HIST1H2AC, HIST1H2BC, HIST1H2BD, HIST1H1C, HIST1H2BK, HIST2H2BE, HIST2H2BF, HIST1H2BJ, HIST3H2A, HIST3H2BB, HIST2H4A, HIST1H4I, HIST1H3H, HIST1H4H
GOTERM_CC_FAT	GO:0016327~apicolateral plasma membrane	15	2.09205	7.43E-05	F11R, PARD6B, CLDN18, CLDN12, EVPL, CGN, MARVELD2, DSG3, PPL, PKP3, PKP4, DSC3, PERP, TJP3, PDZD3
GOTERM_CC_FAT	GO:0030057~desmosome	7	0.97629	1.14E-04	EVPL, DSG3, PPL, PKP3, PKP4, DSC3, PERP
GOTERM_CC_FAT	GO:0016324~apical plasma membrane	16	2.23152	3.87E-04	MUC1, MYO1A, SLC22A18, STX3, CLCA4, ERBB3, SLC5A1, PRKCI, AQP5, DUOX1, LMO7, ABCG8, EZR, RHCG, STK39, SLC39A4
GOTERM_CC_FAT	GO:0032993~protein-DNA complex	12	1.67364	7.95E-04	HIST1H2AC, HIST1H2BC, HIST1H2BD, HIST1H1C, HIST1H2BK, HIST2H2BE, HIST2H2BF, HIST1H2BJ, HIST3H2A, HIST3H2BB, HIST2H4A, HIST1H4I, HIST1H3H, HIST1H4H
GOTERM_CC_FAT	GO:0044427~chromosomal part	30	4.1841	0.00138	HIST1H2AC, CHEK1, HIST2H4A, SPC24, SPC25, HIST1H2BK, OIP5, HJURP, HIST1H2BJ, SKA3, HIST3H2A, HIST1H4I, ASF1B, HIST3H2BB, CDCA5, HIST1H4H, CENPN, HIST1H2BC, HIST1H2BD, MKI67, HIST1H1C, RCOR2, NUF2, CENPF, BIRC5, HMGA2, HMGA1, CENPI, HIST2H2BE, ZWINT, HIST2H2BF, HIST1H3H
GOTERM_CC_FAT	GO:0030054~cell junction	37	5.160391	0.00155	CLDN18, SLC5A1, FERMT1, FHL2, LMO7, GJC2, CLDN12, PCDH1, MARVELD2, PPL, SLC2A1, CHRNA5, PARD6B, F11R, ICA1, STX3, CDHR2, GJB3, GJB5, CAMK2N1, TNS4, SDC1, ARHGAP32, LAMA3, EVPL, ITGA6, DSG3, CGN, PKP3, PKP4, DSC3, CDC42BPA, PERP, TJP3, PDZD3, STEAP1, IGSF9
GOTERM_CC_FAT	GO:0012505~endomembrane system	51	7.112971	0.001314	CYP3A4, CYP3A5, S100A6, HTATIP2, CLTB, SLC22A18, CYP2C18, CYP2S1, NUP62CL, LEMD1, CYP2W1, SPINK5, SLC35A2, AP1S3, APOB, AP1S1, PTGES, ASPH, ELOVL6, RAB27B, JPH1, STX1A, BCL2L14, DUOXA1, TMEM38A, CDHR5, CLIC1, CDS1, ST6GALNAC1, AADAC, ARHGAP32, CHST6, ASPHD2, STEAP2, DOLPP1, AP1M2, MAL2, GIPC1, ECE2, FUT2, GAD1, QSOX1, UPK2, ICA1, CENPF, TMPRSS3, RNF43, CYP4F3, HSPD1, ABO, DMBT1

Table S6(Continued)

List Total	Pop Hits	Pop Total	Fold Enrichment	Bonferroni	Benjamini	FDR
551	329	13528	3.059637	1.25E-06	1.25E-06	9.09E-07
551	414	13528	2.668666	1.20E-05	6.01E-06	8.73E-06
551	91	13528	5.126184	5.03E-05	1.68E-05	3.66E-05
551	220	13528	3.347962	5.25E-05	1.31E-05	3.82E-05
551	220	13528	3.347962	5.25E-05	1.31E-05	3.82E-05
551	224	13528	3.288177	7.88E-05	1.58E-05	5.73E-05
551	229	13528	3.216383	1.29E-04	2.15E-05	9.38E-05
551	565	13528	2.172719	0.001039	1.49E-04	7.55E-04
551	370	13528	2.455172	0.002694	3.37E-04	0.00196
551	199	13528	3.084387	0.00443	4.93E-04	0.003226
551	227	13528	2.920249	0.004442	4.45E-04	0.003235
551	295	13528	2.580012	0.008781	8.01E-04	0.006408
551	81	13528	4.546616	0.010172	8.52E-04	0.007428
551	776	13528	1.86669	0.011264	8.71E-04	0.00823
551	91	13528	4.046987	0.040392	0.002941	0.029951
551	184	13528	2.935532	0.044756	0.003048	0.033261
551	93	13528	3.959956	0.051745	0.003315	0.038595
551	84	13528	4.091954	0.074075	0.004517	0.0559
551	137	13528	3.225774	0.092728	0.005392	0.070676
551	87	13528	3.950852	0.10647	0.005908	0.081757
551	34	13528	6.498986	0.114977	0.006088	0.088701
551	117	13528	3.357501	0.171107	0.008897	0.136252
551	15	13528	9.82069	0.431288	0.025327	0.409205

Table S6(Continued)

List Total	Pop Hits	Pop Total	Fold Enrichment	Bonferroni	Benjamini	FDR
551	45	13528	4.910345	0.624114	0.04165	0.708374
529	190	12782	3.306457	1.01E-04	1.01E-04	3.87E-04
529	147	12782	3.451796	9.28E-04	4.64E-04	0.003558
529	179	12782	3.104688	0.001643	5.48E-04	0.006304
529	99	12782	3.660996	0.01886	0.003801	0.072958
529	63	12782	4.602394	0.016725	0.004208	0.06463
529	102	12782	3.553319	0.026246	0.004423	0.101897
529	20	12782	8.4569	0.04	0.005815	0.156357
529	133	12782	2.906775	0.129312	0.01716	0.52938
529	86	12782	3.371522	0.247866	0.031153	1.085902
529	386	12782	1.87792	0.390156	0.043964	1.877846
529	518	12782	1.725898	0.426052	0.045214	2.105753
529	782	12782	1.57582	0.3755	0.045989	1.788483

Table S7: GO pathway analysis for DEGs of subtype G2 using DAVID.

Category	Term	Count	%	PValue	Genes	
GOTERM_MF_FAT	GO:0004035~alkaline phosphatase activity	3	0.626305	1.11E-04	ALPPL2, ALPI, ALPP	
**List Total**	**Pop Hits**	**Pop Total**	**Fold Enrichment**	**Bonferroni**	**Benjamini**	**FDR**
37	6	12983	175.4459	0.018863	0.018863	0.135901
